# Implantable Bioresponsive Hydrogel Prevents Local Recurrence of Breast Cancer by Enhancing Radiosensitivity

**DOI:** 10.3389/fbioe.2022.881544

**Published:** 2022-04-12

**Authors:** Zhiguang Fu, Hongqi Li, Peng Xue, Hanying Yu, Shuo Yang, Cheng Tao, Wei Li, Yingjie Wang, Jianjun Zhang, Yu Wang

**Affiliations:** ^1^ Department of Tumor Radiotherapy, Air Force Medical Center, PLA, Beijing, China; ^2^ Department of Stomatology, The First Medical Center, Chinese PLA General Hospital, Beijing, China; ^3^ College of Chemical Engineering, Beijing University of Chemical Technology, Beijing, China; ^4^ Department of Oncology, Air Force Medical Center, PLA, Beijing, China

**Keywords:** implantable and enzyme-sensitive hydrogel, sunitinib nanoparticles, breast cancer, radiosensitivity, local recurrance

## Abstract

Breast cancer is one of the most common types of cancer. Patients are often concerned about regional recurrence after breast cancer surgery. Radiotherapy plays a vital role in reducing recurrence and prolonging the survival of patients undergoing breast-conserving surgery and high-risk mastectomy. However, 8–15% of patients still have disease progression due to radiation resistance. Therefore, new strategies for combination radiotherapy sensitization must be investigated. In this study, an implantable drug loading system, sunitinib nanoparticles @ matrix metalloproteinases -response hydrogel (NSMRH), uses enzyme-sensitive hydrogel as a carrier to load sunitinib nanoparticles, was identified. The releasing profile demonstrated that sunitinib nanoparticles may be continuously released from the hydrogels. Functional experiments revealed that, when paired with NSMRH, radiation may significantly inhibit tumor cell proliferation, migration, and invasion *in vitro*. Further animal experiments showed that NSMRH combined with radiotherapy could more effectively control the recurrence of subcutaneous xenograft tumors, prolong the survival time, and have no obvious toxicity in nude mice. Finally, by studying the molecular mechanism of NSMRH, it was hypothesized that in breast cancer cells, NSMRH cooperated with sensitized radiotherapy, mainly due to significantly blocking the G2/M phase, reducing the DNA repair efficiency, inhibiting tumor angiogenesis, promoting apoptosis, and reversing the abnormal expression of platelet-derived growth factor receptor alpha (PDGFRA) after radiotherapy. These findings suggest that NSMRH’s radiation sensitization and anti-tumor activity may aid in the development of a novel method in future clinical applications.

## Introduction

Breast cancer is one of the most widespread and lethal forms of cancer. Breast cancer incidence keeps increasing despite decades of laboratory, epidemiological, and clinical research (Siegel et al., 2021; Sung et al., 2021). From the latest estimates on the global burden of cancer of the International Agency for Research on Cancer (IARC), a notable observation in 2020 is that female breast cancer is now the leading cause of cancer incidence worldwide (https://www.iarc.fr/faq/latest-global-cancer-data-2020-qa/). Radiotherapy (RT) has long been recognized as a key component of the multidisciplinary management of breast cancer (Cao et al., 2021). In properly selected patients, RT not only improves local control, saving patients the morbidity and misery of local recurrence, but it also improves survival, probably by preventing distant metastases from persisting reservoirs of locoregional disease from seeding and reseeding (Maani and Maani, 2021). Nevertheless, local control of the disease still fails in 8–15% of RT-treated patients (Speers et al., 2015). In many cases, locoregional recurrence is thought to be due to the presence or evolution of radioresistant tumor cells and the high invasiveness and metastasis of breast cancer cells (Coates et al., 2015). Therefore, the improvement of radiosensitization and prevention of local recurrence represents an important clinical problem. Meanwhile, a new strategy of combined RT is crucial.

Ionizing radiation has been shown to activate receptor tyrosine kinase (RTKs) to promote cell survival and metastasis, resulting in radiation resistance (Iida et al., 2020; Paranthaman et al., 2020). While small-molecule receptor inhibitors, such as sunitinib, have been suggested as radiosensitizing agents (Brooks et al., 2012; El Kaffas et al., 2013); sunitinib, a potent inhibitor of multiple RTKs, possess antitumor and anti-angiogenic activity against several types of cancer, including breast cancer (Cuneo et al., 2008; Kim et al., 2021). Sunitinib has been shown to have effective radiosensitization action in clinical studies for a variety of cancers due to its strong inhibitory efficacy for RTKs, such as KIT, platelet-derived growth factor (PDGF), and vascular endothelial growth factor (VEGF) receptors (Ghimirey et al., 2021). Nevertheless, such radiosensitizing properties that use sunitinib in combination with irradiation (IR) have not yet been fully studied in breast cancer.

The development of nanotechnology in the biomedical field has opened up a new way for tumor diagnosis and treatment (Kashyap et al., 2021). As radiosensitizers or a carrier of radiosensitizers, it is possible to provide a new opportunity for the further combination of RT (Kempson, 2021; Mortezaee et al., 2021). Hydrogels are 3D networks of cross-linked hydrophilic polymer chains. Injectable biodegradable hydrogels with the ability to generate gels *in situ* have been widely used in biomedical applications such as cell/drug delivery and tissue engineering (Norouzi et al., 2016). To better adapt to the tumor microenvironment and respond to different conditions, some classes of injectable hydrogel demonstrate a sol-gel phase transition upon injection in response to external stimuli, such as temperature, pH, and light (Nguyen and Lee, 2010). Matrix metalloproteinases (MMPs), a family of extracellular enzymes involved in cancer initiation, progression, and metastasis, can degrade the natural extracellular matrix (ECM) and basement membrane, opening up more space for tumor growth (Mura et al., 2013). Due to their ability to be degraded by cell-secreted factors, enzyme-sensitive hydrogels are a promising class of materials for cell encapsulation and tissue engineering (Amer and Bryant, 2016).

**SCHEME 1:**
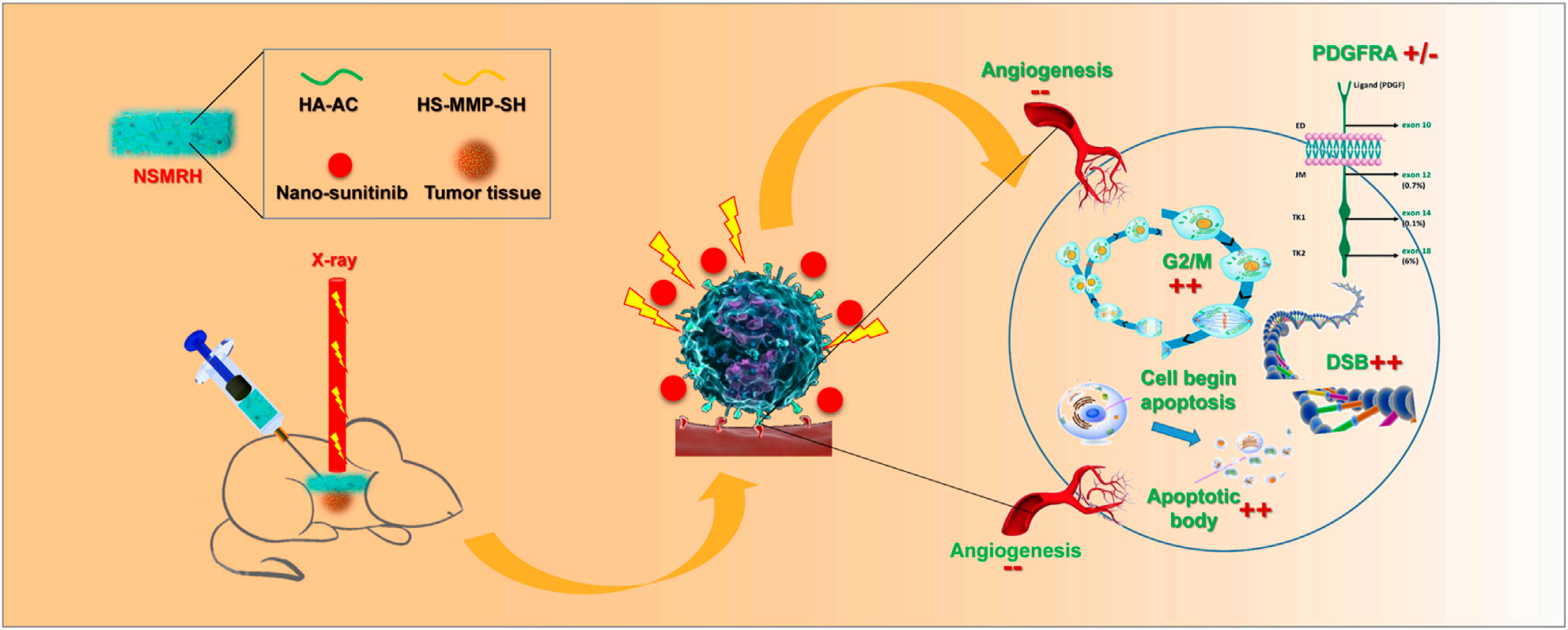
A schematic diagram of the synthesis of sunitinib nanoparticles and its mechanism.

Accordingly, we herein synthesized an MMP-responsive nano complex constructed from hyaluronic acid–acrylate (HA-AC) hydrogel loaded with Sunitinib nanoparticles (denoted as NSMRH) to achieve targeted therapy combined with RT (Scheme 1). In our design, HA-AC was employed as a polymeric network for not only actively targeting tumor cells with high CD44 expression on the cell membrane but also for incorporating sunitinib nanoparticles (nano-su). When NSMRH meets the highly secreted MMPs in the tumor microenvironment, MMPs sensitive polypeptide HS-MMP-SH (GCRDGPQGIWGQDRCG) breaks and the nano-su will gradually release to increase the efficacy when combining with RT, mainly through inducing cell-cycle arrest, DNA damage, and reducing angiogenesis. Such a combination therapy strategy may inhibit the local recurrence and improve the clinical benefit of patients.

## Materials and Methods

### Materials

Sunitinib (99%) and Dihydrazide adipate (99%) were purchased from Aladdin. Peptide crosslinker HS-mmp-SH (99%) was obtained from JenKem Technology. Polyethylene glycol (99%) and D. L-lactide (98%) were purchased fromSigmaa. Hyaluronic acid (injection grade) was obtained from Bloomage Freda. The transwell system was purchased from Millipore (Billerica, MA, United States). MDA-MB-231 cells were purchased from the American Type Culture Collection (Manassas, VA, United States). Other reagents were obtained from the Chemical Reagent Company (Beijing, China).

### Synthesis and Characterization of NSMRH

#### Synthesis Process of PDLLA-PEG-PDLLA

30 g polyethylene glycol (molecular weight 9–10 kDa) and 10 g GD, L-lactide (molecular weight 144 Da) were added into a round bottom flask, and the water is removed by vacuuming at 110°C for 6 h. Then, under the protection of argon, argon was inserted into the round bottom flask to discharge oxygen, and a certain quantity of catalyst stannous octanoate was added into the flask, after mixing, the temperature was changed to 135°C for 24 h. After the reaction product was completely dissolved in dichloromethane, it was precipitated with n-pentane and repeated three times to purify the polymer and remove the unreacted monomer and catalyst; the final product was dried in a vacuum to obtain PDLLA-PEG-PDLLA.

#### Synthesis of Acrylate-Based Hyaluronic Acid (HA-AC)

Briefly, in the presence of 4.0 g (20 mmol) catalyst EDC, hyaluronic acid (2.0 g, 50 kDa, containing 5.28 mmol carboxyl) reacts with 25.0 g (143.5 mmol) adipic dihydrazide (ADH) in water with pH = 4.75 overnight. After the reaction, the mixture was filtered for 5 days by dialysis in ultrapure water (MWCO = 8000 DA). The purified intermediate (HA-ADH) was kept at -20 °C after being lyophilized. Next, 2.0 g HA-ADH was accurately weighed and reacted with n-acryloxysuccinimide (NHS AC) (1.40 g, 8.28 mmol) in HEPES buffer (10 mm HEPES, 150 mm NaCl, 10 mm EDTA, pH 7.2) overnight, then dialyzed in ultrapure water (MWCO = 8000 DA) for 3–4 days, lyophilized and stored at -20°C. Finally, the products were characterized by 1H NMR.

#### Preparation of Sunitinib Nanoparticles by Solvent Precipitation

Take 20 mg of Sunitinib API, dissolve it in 1 ml DMSO, and record it as a drug solution. Then, according to the mass ratio of sunitinib to PDLLA-PEG-PDLLA of1/0, 1/10, 1/20, and1/50, 0 mg, 200 mg, 400 mg, and 1,000 mg PDLLA-PEG-PDLLA were dissolved in PBS (150 mm, pH 7.4). Four parts of a polymer solution containing PDLLA-PEG-PDLLA were obtained as an anti-solvent phase. Add the drug solution into the antisolvent in the ratio of 1/20 of the drug solution and the antisolvent, stir the magneton for 30 min, and record it as the mixture. Dialysis with ultrapure water (MWCO = 3500, 4°C) was performed on the mixture. Sunitinib nanoparticle (nano su) was well prepared after dialysis and freeze-drying.

#### Loading of Nano-Su Into HA-AC

Lyophilized nano-su product was added into PBS (5 ml, pH 7.4) and ultrasonically vibrated for 30 min. Then the nano-su solution was used to dissolve the polymer HA-AC, so as the concentration of HA-AC reached 50 mg/ml, and then the TEA buffer (300 mM, pH 10.13) was used to dissolve the thiol cross-linking agent HS-MMP-SH. The HS-MMP-SH concentration was 50 mg/ml. Subsequently, the two solutions were mixed with a volume ratio of 4/1 to solidify the drug-loaded water gel, which required about 5 min from the solution state to the solidification state.

#### Morphology, Particle Size, and Stability of Nano-su

Ultrasonic dispersion for 5 min was used to prepare TEM samples. The morphology of the samples was observed by TEM. Place samples in a dark place for 1 week to observe their stability.

#### *In vitro* NSMRH Release Assay

250 uL NSMRH (10 mg/ml nano-su) was immersed into a tube containing 20 ml of PBS, with or without MMP-2 (1.5 μg/ml) and transferred into a dialysis bag (MWCO = 3500 Da). At predetermined time intervals, 3 ml of release medium was withdrawn from the tube and replaced by 3 ml of fresh medium. The release of nano-su was measured by determining the absorption of the samples at 485 and 795 nm, respectively.

### Cell Viability Assay

Specifically, cells were first seeded in 96-well plates (5.0 × 10^3^ cells/well) with 100 μL media and incubated at 37°C overnight. Then, the culture medium was removed and 20 μL of the hydrogel precursor solutions, including MMP-response hydrogel (MRH, 4% HA-Ac), NSMRH (12.5 μg/ml, 25 μg/ml, 50 μg/ml, and 100 μg/ml nano-su) for 48 h, with or without IR (4 Gy given as a single fraction using a 60 Co. source at 3.3 Gy/min at room temperature). After incubation, 10 μL CCK-8 reagent was added to each well, and the cells were incubated at 37°C for 2 h. The plates were read on a microplate reader (OD450) after being shaken thoroughly.

### EdU Incorporation

For EdU labeling, a 1:1,000 dilution of EdU-labeling reagent (Invitrogen) was added to the islet culture medium during the last 18 h of cell culture. EdU was detected using the Click-iT kit (Invitrogen) following the manufacturer’s protocol. The slides were counterstained with DAPI (4′, 6-diamidino-2-phenylindole).

### Colony-forming Assay

Cell survival was determined by a standard colony-forming assay. Exponentially growing cells were seeded into dishes (150 cells per dish) and treated with different doses of radiation (The doses applied in the experiments varied from 0 to 8 Gy), with or without NSMRH. After being cultured for 10–15 days, colonies in the dishes were stained with 0.5% crystal violet for 30 min. The plating efficiency was determined by counting the number of colonies with >50 cells. Plating efficiency (PE) = colonies observed/cell planted ×100%. Surviving fraction = PE/PE of the nonirradiated group.

### *In vitro* Scratch Assay

A scratch assay was performed to evaluate the mobility of the breast cancer cells. MDA-MB-231 cells were seeded onto 24-well plates (5 × 10^3^ per well). After 24 h of culture, each well was manually scratched with the tip of a pipette. The NSMRH precursor solutions were added to the wells. After the hydrogels were formed at 37°C, a fresh culture medium was added to the wells and the cells were treated with or without IR. The scratch area was photographed at given time points (0 and 24 h), and the relative migration distances between two cell edges were analyzed using the ImageJ software (NIH).

### Invasion Assay

For the transwell invasion assay, 24-well transwell units with an 8-μm pore size polycarbonate (Millipore) were used according to the manufacturer’s instructions. Briefly, filters were coated with Matrigel (BD, Bedford, United States) to form a continuous, thin layer. After irradiation, the cells were incubated for 6 days. Equal numbers of irradiated cells or control cells (1 × 10^5^/well) were plated in the upper chamber in a serum-free medium, with or without treatment of NSMRH (100 μg/ml nano-su); in the lower chamber, 10% FBS medium was added. Cells were fixed after 24 h with 4% paraformaldehyde, stained with crystal violet and counted.

### *In vivo* Anti-recurrence Efficacy of NSMRH

BALB/c female nude mice (4–6 weeks old, weight 18–20 g) were purchased from Vital River Laboratory Animal Technology Co. Ltd. (Beijing, China). All animal procedures were performed in compliance with the Guidance Suggestions for the Care and Use of Laboratory Animals and approved by the Institutional Animal Care and Use Committee of Beijing Vital River Laboratory Animal Technology Co. Ltd. To determine the anti-recurrence efficacy of NSMRH *in vivo*, a subcutaneous tumor recurrence model was established by using nude mice. MM-231 cells were incubated subcutaneously in the right flank of female nude mice (6–8 weeks). On day 15, we resected around 90% of total tumors (∼500 mm^3^) and randomly divided mice into three groups (*n* = 5) immediately: (a) implantation of MRH; (b) implantation of MRH + RT; (c) implantation of NSMRH + RT. During treatment, boy weights and tumor volumes were measured every 2 days, and the volume was calculated as tumor volume (mm^3^) = (length)*(width) ^2^/2. Survival rates were compared using Kaplan–Meier survival curves.

### 2.9 Histological Assay

Tumors and main organs (brain, heart, liver, spleen, lungs, and kidneys) collected from the mice were fixed in 4% paraformaldehyde. Resected tumor tissue was stained for TUNEL apoptosis assay (Roche, Beijing, China) and immunohistochemistry assay of caspase3 (Santa Cruz Biotechnology, United States) according to the manufacturer’s protocols. Tissue sections of major organs were stained with H&E to observe histopathological changes.

### Flow Cytometric Cell Cycle Analysis

After different treatments, cells were harvested by trypsinization at the 90% confluent stage, fixed with 70% ethanol at 4°C overnight, and stained with a solution containing propidium iodide (PI) and DNase-free RNase, according to the manufacturer’s instructions. Cells were then run on a Becton Dickinson FACScan flow cytometer and analyzed for PI fluorescence intensity. The relative proportions of cells in the G1, S, and G2/M phases of the cell cycle were determined from the flow cytometry data.

### Immunofluorescence of γ-H2AX

Cells were treated under different conditions and then exposed to X-ray irradiation (5 Gy). For the detection of γ-H2AX foci, the cells were fixed after 3 h of X-ray irradiation. The cells were treated with phosphate-buffered saline (PBS; 6.7 mM, pH 7.4, NaCl 137 mM) containing 1% Triton-X 100, blocked with 3% BSA, and incubated with mouse anti-human γ-H2AX. Fluorescence images of the cells were acquired using a confocal laser-scanning microscope.

### RNA Extraction, Reverse Transcription and Real-Time Quantitative Polymerase Chain Reaction

Total RNA was extracted using the TRIzol Reagent (OMEGA Bio-Tek). Reverse transcription was performed using the PrimeScript RT Reagent Kit (TaKaRa Biotechnology). All primers including PDGFRA, PDGFRB, FLT1, PDGFRL, and GAPDH were synthesized by Shanghai Sangon Co. (Sangon, Shanghai, China) Real-time PCR was performed using the SYBR Premix Ex Taq II Kit (TaKaRa Biotechnology).

### Western Blot Analysis

After cell attachment, the different conditioned media was collected. Tumor samples were minced on ice in pre-chilled lysis buffer containing phenylmethylsulfonyl fluoride, protease inhibitors, and phosphatase inhibitors (KeyGen Biotech, China). Homogenized tissues and cell lysates were then centrifuged at 14,000 rpm at 4°C for 15 min. Immunodetection was performed using a Western-Light chemiluminescent detection system (Applied Biosystems, Foster City, CA, United States) after incubation with the secondary antibody for 1 h.

### Statistical Analysis

Statistics were evaluated using GraphPad Prism V8.0 software (GraphPad Software, La Jolla, CA, United States). Each experiment was repeated at least three times. Statistical analysis was carried out using one-way ANOVA (multiple comparisons) and Student’s t-test (two comparisons, or two-tailed). Differences were deemed significant if *p* < 0.05. (*** indicates *p* < 0.001, ** indicates *p* < 0.01, * indicates *p* < 0.05, and #indicates *p* > 0.05 in Figures). Survival rates were compared using Kaplan–Meier survival curves.

## Results

### Sunitinib Nanoparticles Were Successfully Prepared and Showed Good Physicochemical Properties

**FIGURE 1:**
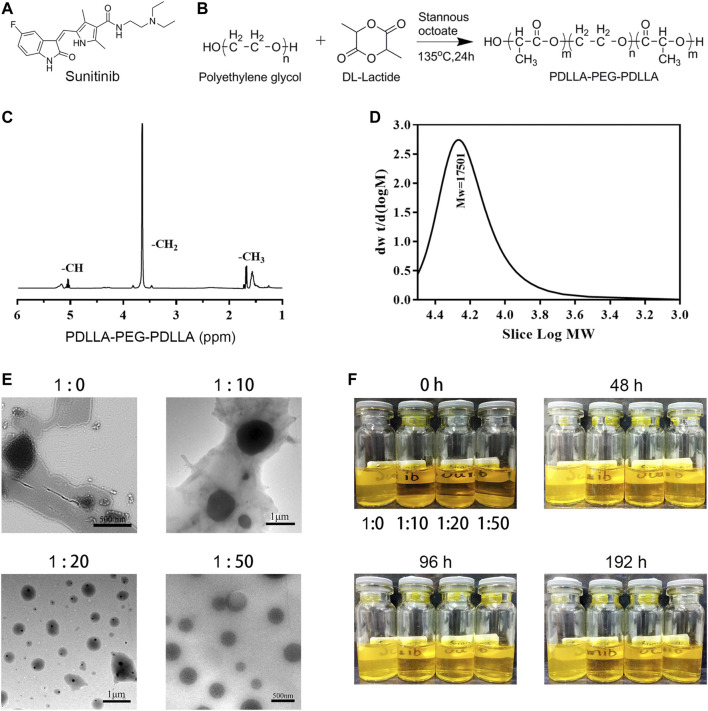
Preparation and characterization of sunitinib nanoparticles. **(A)** Chemical structure of sunitinib. **(B)** The synthesis process of PPP. **(C)** HNMR of PPP. **(D)** Gel permeation chromatography (GPC) of PPP. **(E)** Representative SEM image of the nano-su. **(F)** Photograph of nano-su mixture solution at room temperature.

Considering sunitinib malate has a relatively large molecular weight (532.56 Da) and poor water solubility (water<1 mg/ml), such a molecular structure and physicochemical properties make it difficult to be loaded in hydrogels (Figure 1A). Therefore, sunitinib nanoparticles should be synthesized essentially. After continuous screening and verification (data not shown) of the appropriate surface-modifying reagent, an amphiphilic diblock (hydrophilic hydrophobic) copolymer, PDLLA-PEG-PDLLA (PPP) was chosen, for the preparation of nano-su (Figures 1B–D). By physical encapsulation, the hydrophobic block of PPP was contained on hydrophobic drugs (sunitinib). While the hydrophilic block was distributed around the drugs, resulting in the formation of a hydrogen bond, which stretches to the water to form a shell with a certain thickness. Then nano-su was prepared according to the mass ratio of different sunitinib/PPP. From the TEM image (Figure 1E), it was noticed with the increasing proportion of PPP, the morphology of nano-su tended to be regular, round, and spherical. The particle size of nano-su was about 400 nm when the mass ratio of sunitinib/PPP reached 1/50. Finally, we discovered that the water dispersion stability of nano-su was highest when the mass ratio was1/50, although varying degrees of precipitation emerged in samples under other conditions (mass ratio1/0, 1/10, and 1/20) (Figure 1F). To the best of our knowledge, this is the first time to prepare sunitinib nanoparticles. It was difficult for nanoparticles to interact and aggregate, with the help of good water solubility and a large exclusion volume of PEG. Meanwhile, the repulsion between hydrophilic segments can assure the long-term survival of drug nanoparticles in a specific concentration range. The relevant contents have been authorized to the Chinese national utility model patent (CN 201711358718.8).

### Morphology and Characterization of NSMRH

**FIGURE 2:**
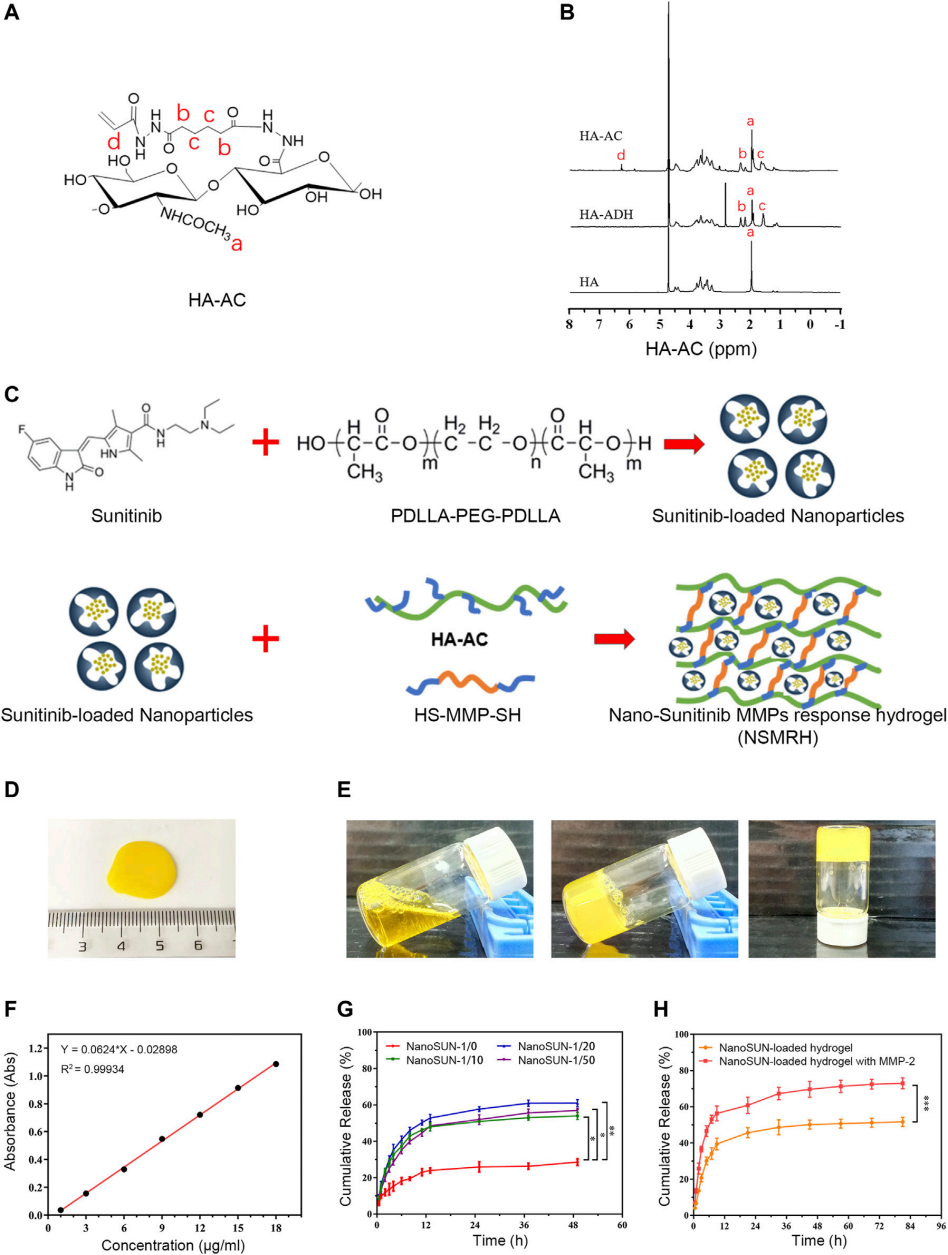
Morphology and characterization of NSMRH. **(A)** Chemical structure of HA-AC. **(B)** HNMR of HA-AC. **(C)** process Schematical process of NSMRH. **(D)** General morphological map of NSMRH. **(E)** Left: Photograph of the mixture solution at room temperature. Middle and right: Photograph of the as-prepared hydrogels formed in 5 min at 37°C. **(F)** Dissolution standard curve of nano-su. **(G)** and **(H)** Nano-su release profile of NSMRH at various conditions.

Our previous work has fully demonstrated that MMP-responsive hydrogels, as a suitable delivery carrier, successfully loaded anti-tumor drugs to achieve the combined therapeutic effect of tumor (Wang et al., 2019). In this investigation, we attempted to load nano-su with this carrier. HA-AC was well prepared (Figures 2A,B), with a focus on previous work. After loading of nano-su into the MRH, NSMRH was well synthesized and presented generally spherical (Figures 2C,D). By inverting tube test, we observed that it takes about 5 minutes for the mixture to change from liquid to solid, which tended to be stable (Figure 2E). Finally, we evaluated the drug dissolution of NSMRH *in vitro*. The dissolution standard curve was well prepared (Figure 2F), and the dissolution curve of nano-su in PBS was shown in Figure 2G. We discovered that adding PPP to the reaction system improved the dissolution rate of nano-su. Specifically, the cumulative drug dissolution rate of samples with a mass ratio of 1/0 was only 28%, while the dissolution rate of samples with a mass ratio of 1/20 and 1/50 reached about 60%, at 48 h. In addition, Figure 2H showed that the dissolution rate of hydrogels was faster and higher in the absence of the MMP-2 enzyme, indicating the intelligent response characteristics. In 72 h, for example, nano-su released less than 50% without the addition of MMP-2 enzyme, but more than 70% when it was added.

### NSMRH Inhibits the Activity of MM-231 Cells and Promotes the Radiosensitivity *in vitro*

**FIGURE 3:**
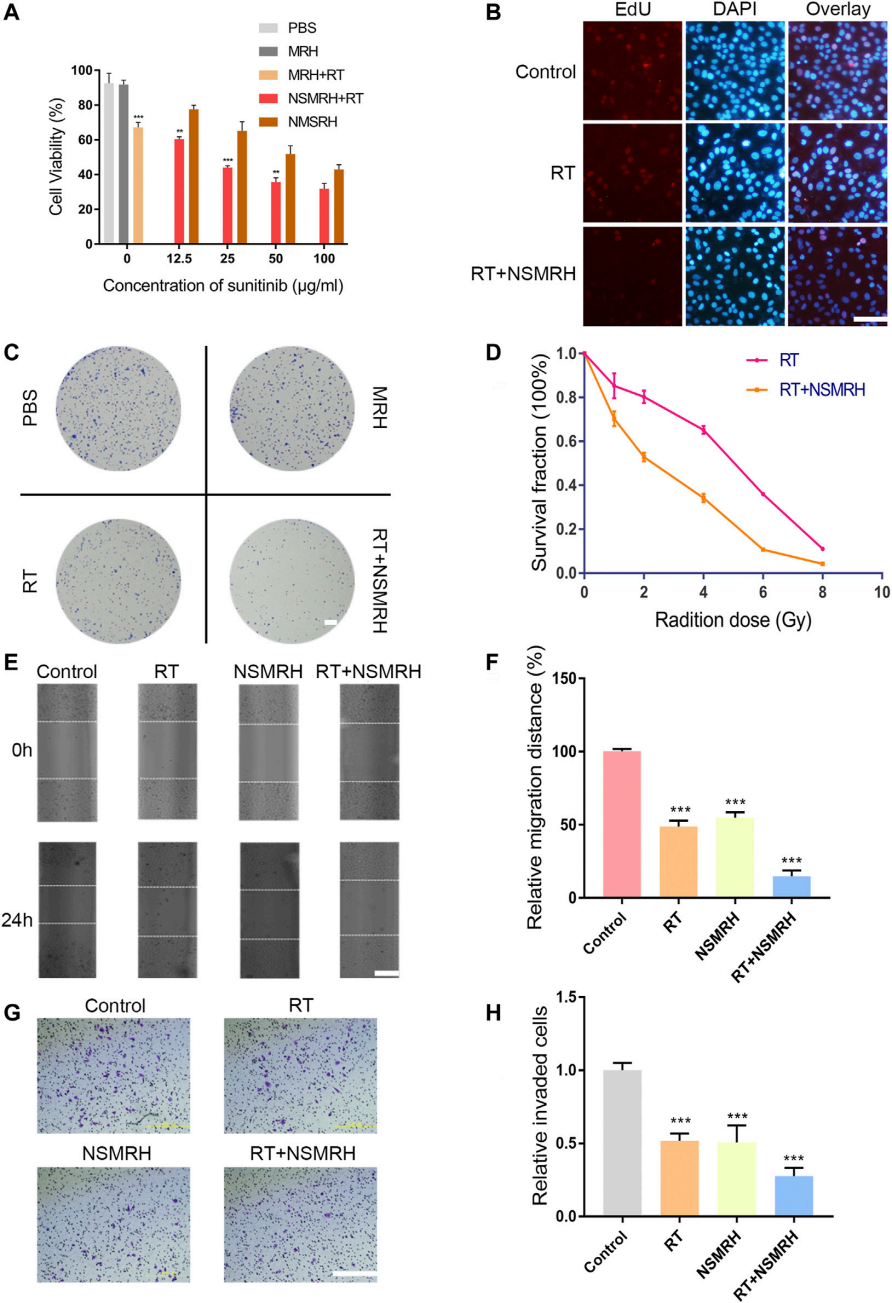
*In vitro* functions of NSMRH in MM-231 cells. **(A)** Effects of different conditions on MM-231 cells viability at 48 h. **(B)** Edu staining of MM-231 cells, red fluorescence represents proliferating cells, scale bars: 100 μm. **(C)** A clonogenic assay was performed. Colonies larger than 50 cells were counted, and phase-contrast images were taken, scale bar: 2.5 mm. **(D)** Survival curves of MM-231 cells exposed to increased irradiation. **(E)** Effect of NSMRH on MM-231 cells migration. Representative images showing tumor cells migration, scale bars: 100 μm. **(F)** The figure shows quantitative analysis of a wound healing assay, with triplicate measurements of three independent experiments. **(G)** Photomicrographs illustrate representative fields of invaded cells, scale bars: 100 μm. **(H)** The relative number of invaded cells was calculated, and the data are presented in a histogram from three independent experiments.

To investigate the antitumor therapeutic effects of combination therapy *in vitro*, a CCK-8 assay was performed 48 h after exposure to various concentrations of nano-su. PBS or MRH alone did not inhibit the viability of MM-231 cells after 48h, indicating good biological safety of the hydrogel (Figure 3A). Furthermore, as compared to the control, NSMRH significantly suppressed tumor cell proliferation in a dose-dependent manner. Notably, the impact of NSMRH and IR combination treatment was also studied. For each concentration, the cell growth inhibition by combination treatment was significantly higher than either NSMRH or IR, separately (Figure 3A). For Edu staining (Figure 3B), comparing the control group and RT group, the intensity of red fluorescence in the combination group was markedly reduced, suggesting proliferating cell reduction. Next, we performed a clonogenic assay to assess the potential radiosensitization activity of NSMRH (Figures 3C,D). After exposure to X-ray irradiation at doses varying from 0 to 8 Gy, the survival fractions of cancer cells were determined. From Figure 3D, we could easily observe survival fraction (SF) of the combination treatment group was much lower than the RT treatment alone, especially when the does came to 4 Gy. Furthermore, clones created by combination treatment contained fewer cells than clones formed by RT or NSMRH alone, signifying slower cell division. These findings show that NSMRH has radiosensitizing effects on MM-231 cells. Further, we evaluated the effect of combination therapy on the motility of MM-231 cells using an *in vitro* scratch assay. As shown in Figures 3E,F, each single treatment group could inhibit the migration of tumor cells to a certain extent. The group that was incubated with RT + NSMRH, on the other hand, had the highest migration inhibitory distance. Results showed that the combination treatment group significantly inhibited approximately 75% of the migration efficacy compared with the control. Finally, to confirm whether NSMRH could help RT impair the invasive ability of MM-231 cells *in vitro*, a trans-well invasion assay was performed. And we drew similar conclusions, as expected. Compared with the treatment of a single factor, the combined treatment group represented the strongest invasive inhibition of MM-231 cells (Figures 3G,H). Notably, from Figure 3A, we also found there were no obvious effects on cell viability when cells were treated with NSMRH and NSMRH + RT at a maximum concentration of 100 μg/ml. These results implied that the different inhibition rate between NSMRH and NSMRH + RT was not due to cytotoxicity. All of the above findings showed that NSMRH may not only reduce the survival of MM-231 cells but also effectively enhance their radiosensitivity.

### NSMRH Sensitizes Local RT and Effectively Inhibits Tumor Recurrence

**FIGURE 4:**
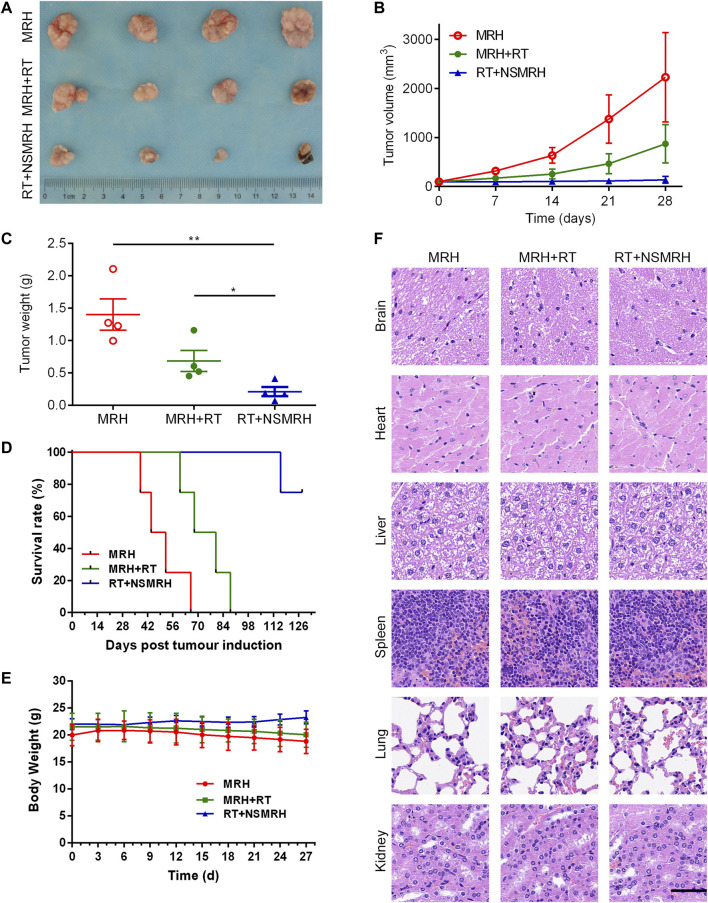
NSMRH inhibits tumor recurrence *in vivo*. **(A)** *in vivo* tumor tissue imaging about recurrence of subcutaneously transplanted tumor in mice after resection. **(B)** Tumor growth curves of different groups up to 28 days. **(C)** Tumor weight curves of different groups. **(D)** The survival rates of nude mice after treatment were illustrated by Kaplan–Meier curves. **(E)** Bodyweight changes of the mice during treatments. **(F)** Histological sections of the main organs (brain, heart, liver, spleen, lungs, and kidneys) were stained by H&E, scale bars: 100 μm.

A subcutaneous breast tumor recurrence model (an incomplete tumor resection) was established to assess the efficacy of combination therapy *in vivo*. Surgery was performed with ≈10% tumor tissue left intentionally on day 15, after receiving conventional-dose local irradiation, a blank hydrogel (MRH), and NSMRH was placed into the tumor resection cavity. As shown in Figure 4A, mice in the control, MRH group were exhibited with nearly entire local rapid regrowth. In comparison with the control group, the MRH + RT group decreased the recurrence rate to 60% within 27 days. While the combination treatment significantly reduced local tumor recurrence, it also inhibited approximately % of the residue MM-231 cells. Meanwhile, the tumor volume and tumor weight in mice treated with NSMRH + RT were much smaller and lighter than that of other groups (Figures 4B,C). Furthermore, RT + NSMRH treatment considerably increased median survival time (Figure 4D). In a toxicity test, no obvious variations in mice weight during any of the treatments (Figure 4E). Through H&E staining, combination therapy didn’t affect other major organs, either (Figure 4F). The results above indicated NSMRH increased the radiosensitivity, especially leading to the inhibition of local recurrence *in vivo*.

### Possible Mechanism of NSMRH Radiosensitization

**FIGURE 5:**
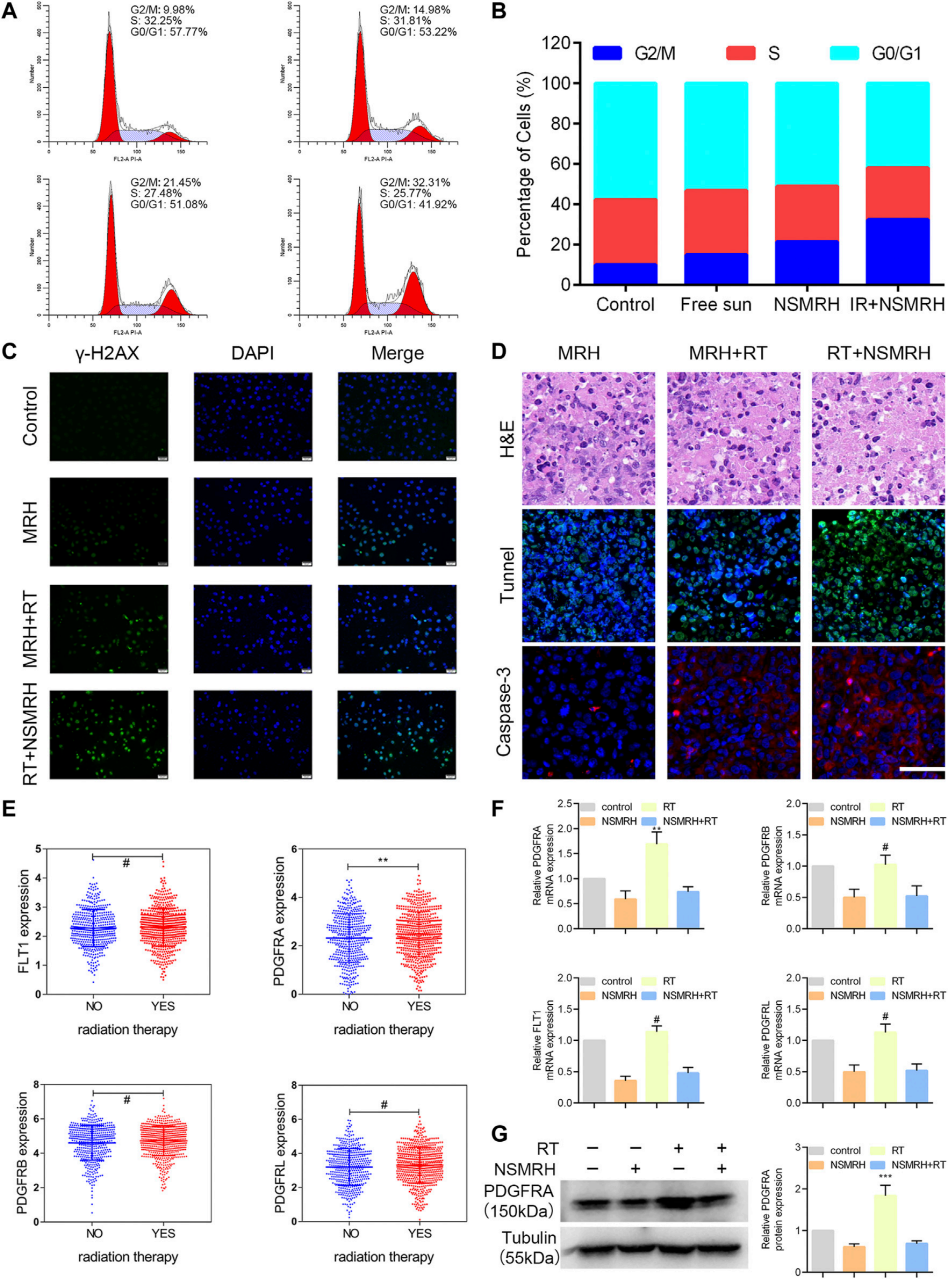
Analysis of the possible mechanism of NSMRH. **(A, B)** NSMRH combined with RT induces G2/M checkpoint of MM-231 cells. FFlowcytometricanalysis of the DNA content in four groups cultured for 24 h. Pictures were representative flow cytometry plots of three experiments. **(C)** NSMRH impairs DSB repair after irradiation in MM-231 cells. DSB repair assay was performed by counting phospho γ-H2AX foci. DSBs were stained as green and the nuclei as blue, 200 ×, scale bars: 50 μm. **(D)** Tunnel and Caspase-3 expression in original orthotopic tumors, as determined by IHC staining analysis. Each inset shows images obtained at × 100magnification, scale bars: 25 μm. **(E)** The mRNA level of PDGFRA, FLT1, PDGFRB, and PDGFRL were analyzed in the TCGA dataset. **(F)** Relative mRNA expression of PDGFRA, PDGFRB, FLT1, PDGFRL, and GAPDH was detected using RT-qPCR in different treatments. **(G)** Left, the protein level of PDGFRA, FLT1, PDGFRB, and PDGFRL were examined by western blot assay; Right, quantification of the grayscale analysis in three independent experiments.

To investigate the mechanisms behind NSMRH-induced radiosensitivity in MM-231 cells, cell cycle distribution was first assessed by PI staining. Figures 5A,B showed the relative proportion of cells at the G0/G1 phase, S phase, and G2/M phase of the cell cycle. It is no doubt that an accumulation of MM-231 cells in the G2/M phase was noted in combination treatment groups, coupled with a decrease of the G0/G1 proportion. Interestingly, we found that, in the absence of RT, both free sunitinib and nano-su could enhance the G2/M ratio to some extent. The fraction of G2/M that increased following NSMRH therapy was significantly higher than the proportion of G2/M that increased after free sunitinib. It was probably due to the smaller particle size and more uniform distribution of nano-su, which makes the cancer cell more easily uptake. DNA double-strand break (DSB) is the most common change in cells after radiation. To determine if the radiosensitizing impact of NSMRH on MM-231 cells is caused by a defect in DSB repair, the levels of phospho- γH2AX foci at 12 h of 4Gy irradiation were determined by immunofluorescence (Figure 5C). The combination treatment (NSMRH + RT) increased the formation of phosphor-γH2AX foci compared to irradiation alone. These results indicated that NSMRH markedly increases the induction and persistence of IR-induced γ-H2AX foci. Then, we performed immunofluorescence staining of tumor tissues *in vivo* to mainly detect the apoptosis-related molecules. Through the staining of TUNEL and Caspase-3, it was found that NSMRH can significantly promote the apoptosis of tumor cells based on RT (Figure 5D). We also examined both caspase-3 expression and cleaved caspase-3 expression by western blot, as shown in Supplementary Figure S1, with the increase of apoptosis cells, the expression of cleaved caspase-3 increased in total lysates of original orthotopic tumors. Finally, it was worth noting that studies have revealed the targets of sunitinib are mainly members of the VEGFR family and PDGFR family, which play a vital role in tumor progression (Saleh et al., 2020). An interesting result was obtained by analyzing gene expression data in TCGA databases. Patients who received RT had a higher level of PDGFRA mRNA expression than those who did not, whereas FLT1, PDGFRB, and PDGFRL did not change obviously (Figure 5E). Then, using quantitative real-time PCR and western blot, we discovered that both the mRNA and protein levels of targeted molecules in MM-231 cells showed the same trend. Appropriately, a combination of NSMRH was employed, which can greatly inhibit PDGFRA expression and counteract the overexpression generated by RT (Figure 5F). To sum up, we declared NSMRH can enhance the sensitivity of RT, mainly due to amplifying the cell cycle arrest of G2/M, DSB effect, and apoptosis after RT, and reversing the abnormal overexpressed of PDGFRA after receiving IR.

## Discussion

We identified NSMRH, a novel intelligent hydrogel drug loading technology, in this investigation to sensitize RT and accomplish the synergistic impact of combined therapy. NSMRH can be successfully combined with RT to prevent local recurrence and metastasis by being implanted locally in the resection site of a breast tumor and steadily producing nano-su with high MMP secretion. Local recurrence and distant metastasis receiving RT in breast cancer become major causes for tumor progression (Li W et al., 2021; Haussmann et al., 2021). The molecular mechanism may be as follows: (1) the remnants of tumor tissue gradually proliferated due to neovascularization (Giatromanolaki et al., 2002; Arora and Cascella, 2021); (2) The subsets of cancer cells might be resistant to radiation (Bernichon et al., 2017; Mal et al., 2020). Sunitinib is an anti-angiogenic drug that inhibits the activation of a broad range of receptors, including vascular endothelial growth factor (VEGF) receptors. FDA has approved sunitinib for the treatment of renal cell carcinoma and gastrointestinal stromal tumors (Ebos et al., 2007; Brooks et al., 2012). In addition, sunitinib also shows activity in clinical studies of neuroendocrine tumors, NSCLC, colon cancer, primary liver cancer, and breast cancer (Ang et al., 2020; Li H et al., 2021; Chen et al., 2021; Grande et al., 2021; Symonds et al., 2021). What’s more, sunitinib also has been suggested as a radiosensitizing agent (Curigliano et al., 2013; El Kaffas et al., 2013; Affolter et al., 2017). However, we should note that sunitinib was utilized as a systemic medication in the aforementioned studies, either orally (clinical studies) or intravenously (preclinical studies). While in this study, nano-sunitinib was firstly synthesized to load into the hydrogels and for local administration. Results indicated that comparing traditional preparation of sunitinib, nano-su showed ideal physicochemical properties, that make it greatly help to increase the effect of local anti-tumor angiogenesis, enhance the local sensitization of RT, to prevent local recurrence. Meanwhile, loading targeted drugs into the hydrogel would reduce the side effects and increase their biological safety through sustained and controlled release.

Due to the existence of RT resistance, the impact of RT on tumor tissue is insufficient. The discovery of effective and safe RT sensitizers boosts the therapeutic impact significantly. So far, the application of RT sensitizers mainly includes electrophilic nitrogen-containing heterocycles (Adams, 1973; Yamazaki et al., 2013; Zeng et al., 2013), cyclooxygenase-2 inhibitors, related signal pathway inhibitors, and so on (Klenke et al., 2011; Phelps et al., 2014). Notably, nanostructured RT sensitizers have received considerable interest in recent years due to their ability to precisely cluster in tumor tissues, efficiently increase RT sensitivity, and even monitor the local dosage of RT (Babaei and Ganjalikhani, 2014; Chastagner et al., 2015). Currently, these nanoscale radiosensitizer used in breast cancer is still rare. Through this study, it has been demonstrated the effect of NSMRH on enhancing the sensitivity of RT in breast cancer and the function of preventing recurrence and metastasis combined with RT. We also further clarified its molecular mechanism of RT sensitization, mainly including the following aspects: (a) Regulate cell cycle detection and remove the cell cycle resistance caused by RT. (b) Reduce the DNA repair efficiency of tumor cells and increase radiosensitivity. (c) Promote tumor cell apoptosis and directly increase the benefit of RT. (d) Reverse the tumor progression caused by the increased expression of PDGFRA caused by RT. Remarkably, in addition to breast cancer, NSMRH may have potential implications in the treatment of other solid tumors, according to our continuing study. Furthermore, the sensitization impact and mechanism of NSMRH under different RT modalities (such as SBRT et al.) must be investigated further in future research. In conclusion, our findings reveal a promising therapeutic application of NSMRH. Such an approach may have benefits in reducing local recurrence and distant metastasis when in combination with RT. Such radiation sensitization characterization may be worthy of being expanded to clinical practice.

## Data Availability

The original contributions presented in the study are included in the article/Supplementary Material, further inquiries can be directed to the corresponding authors.

## Supplementary Material

Supplementary Figure S1 Expressions of caspase 3 and cleaved caspase 3 in total lysates of original orthotopic tumors from each group.
