# Supplementary figures and images for: Implantable Bioresponsive Hydrogel Prevents Local Recurrence of Breast Cancer by Enhancing Radiosensitivity
Source: Front Bioeng Biotechnol. 2022 Apr 12;10:881544. doi: 10.3389/fbioe.2022.881544 (PMC9039615; doi:10.3389/fbioe.2022.881544)

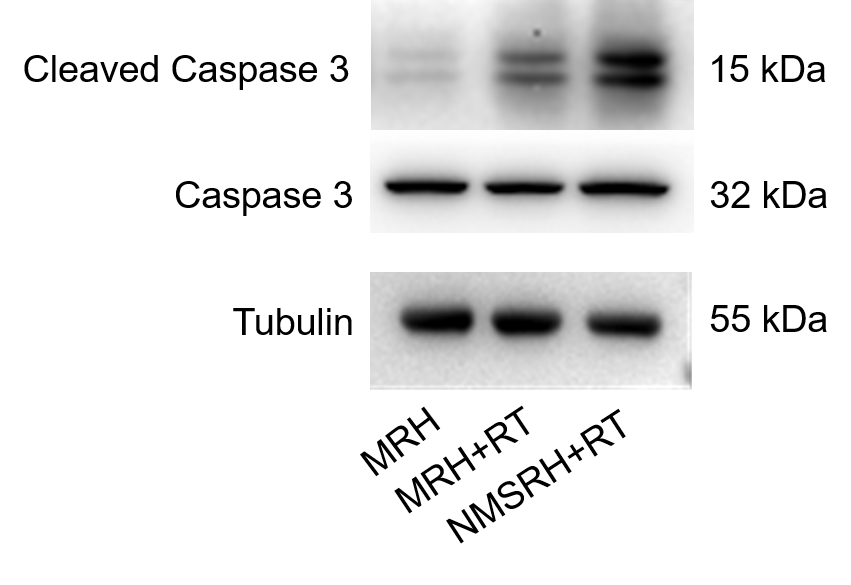

Supplement: Supplementary file 1 [file Image1.TIF]
